# Long-term monitoring data on *Popilliajaponica* (Newman, 1838) (Coleoptera, Rutelidae) across the Azorean Islands

**DOI:** 10.3897/BDJ.12.e138989

**Published:** 2024-12-31

**Authors:** Mário Brum Teixeira, António Onofre Soares, David H. Lopes, Lucas Lamelas-Lopez, Paulo A. V. Borges, José Adriano Mota, Nelson Simões

**Affiliations:** 1 University of the Azores, Biotechnology Centre of Azores (CBA), Faculty of Sciences and Technology, PT-9500-321, Ponta Delgada, Azores, Portugal University of the Azores, Biotechnology Centre of Azores (CBA), Faculty of Sciences and Technology, PT-9500-321 Ponta Delgada, Azores Portugal; 2 University of the Azores, cE3c – Centre for Ecology, Evolution, and Environmental Changes, Azorean Biodiversity Group, CHANGE – Global Change and Sustainability Institute, Faculty of Sciences and Technology, PT-9500-321, Ponta Delgada; Azores, Portugal University of the Azores, cE3c – Centre for Ecology, Evolution, and Environmental Changes, Azorean Biodiversity Group, CHANGE – Global Change and Sustainability Institute, Faculty of Sciences and Technology, PT-9500-321 Ponta Delgada; Azores Portugal; 3 University of the Azores, cE3c – Centre for Ecology, Evolution and Environmental Changes, Azorean Biodiversity Group, CHANGE – Global Change and Sustainability Institute, School of Agricultural and Environmental Sciences, Rua Capitão João d´Ávila, Pico da Urze, 9700-042, Angra do Heroísmo, Azores, Portugal University of the Azores, cE3c – Centre for Ecology, Evolution and Environmental Changes, Azorean Biodiversity Group, CHANGE – Global Change and Sustainability Institute, School of Agricultural and Environmental Sciences, Rua Capitão João d´Ávila, Pico da Urze, 9700-042 Angra do Heroísmo, Azores Portugal; 4 IUCN SSC Atlantic Islands Invertebrate Specialist Group, PT-9700-042, Angra do Heroísmo, Azores, Portugal IUCN SSC Atlantic Islands Invertebrate Specialist Group, PT-9700-042 Angra do Heroísmo, Azores Portugal; 5 IUCN SSC Species Monitoring Specialist Group, PT-9700-042, Angra do Heroísmo, Azores, Portugal IUCN SSC Species Monitoring Specialist Group, PT-9700-042 Angra do Heroísmo, Azores Portugal; 6 Direção Serviços de Agricultura, Direção Regional de Agricultura, Secretaria Regional de Agricultura e Desenvolvimento Rural, Quinta de São Gonçalo, PT-9500-343, Ponta Delgada, Azores, Portugal, Portugal Direção Serviços de Agricultura, Direção Regional de Agricultura, Secretaria Regional de Agricultura e Desenvolvimento Rural, Quinta de São Gonçalo, PT-9500-343 Ponta Delgada, Azores, Portugal Portugal

**Keywords:** agriculture, dataset, Ellisco traps, invasive species, islands, Japanese beetle, monitoring

## Abstract

**Background:**

The Japanese Beetle, *Popilliajaponica* Newman, 1838 (Coleoptera, Rutelidae), is a univoltine agricultural pest that poses a serious threat to various agricultural crops. For more than 16 years, the Azorean official authorities have implemented a Long-Term Ecological Research (LTER) programme that is crucial for understanding the dynamics of insect pests, such as the Japanese Beetle, and their impacts on agricultural ecosystems. The significance of this long-term monitoring extends beyond understanding the pest's life cycle. By sharing this long-term monitoring data with the scientific community, we hope it allows for a more comprehensive assessment of *P.japonica* success and spread, enabling a deeper understanding of how this pest interacts and correlates with biotic and abiotic factors to uncover patterns and trends crucial for effective pest management.

In the Azores Archipelago, *P.japonica* adults emerge from pupae at the end of May and peak in density by early July, persisting until October. Larvae develop through three instars, with the third instar grub pupating by early May. This lifecycle highlights the pest population's seasonal activity, including the timing of emergence, adult stages and larval stages. It reveals when and for how long the pest is active in each of its life stages and provides critical information for pest management strategies. Worldwide, this pest can cause damage to 414 plant species across 94 families, underscoring the potential for elevated crop damage. This information is invaluable for developing targeted intervention strategies and mitigating economic losses caused by *P.japonica* infestations. Therefore, establishing and maintaining long-term programmes for monitoring *P.japonica* populations are essential for scientific understanding and practical pest management efforts in the Azores Archipelago.

**New information:**

The data presented here report the officials' records of a 16-year (from 2008 to 2023) long-term monitoring programme on *P.japonica* in the Azores Archipelago, undertaken by the Secretaria Regional da Agricultura e Alimentação operational services. Based on the last checklist of Azorean arthropods, the species is recorded for the first time for the Corvo, Graciosa and São Jorge Islands.

## Introduction

The Japanese beetle *Popilliajaponica* Newman, 1838 (Coleoptera, Rutelidae) was introduced on Terceira Island (Azores) early in the 1970s (Simões and Martins 1985, Martins et al. 1988, [Bibr B11832174][Bibr B11832371], Martins et al. 1988, Guimarães 1972). Mild temperatures, high relative humidity and high precipitation created the perfect conditions for the beetle's establishment and rapid spread. Despite initial efforts to control the species, it quickly dispersed and threatened local crops, especially pasture lands (Martins et al. 1988, Lopes 1992).

In the Azores Archipelago, *P.japonica* adults appear in the last week of May, reach their peak in the middle of July and disappear in the middle of October. The females can lay up to 60 eggs, from which the first-instar larvae begin to hatch in the second half of July. The 1^st^ instar larvae do not last long and the second instar has a lifespan of between 1 and 5 months. Second-instar larvae feed on fine roots and organic matter at 2.5-5 cm below the ground until early December. The 3^rd^ instar is the one that lasts longest, about 6 to 8 months. Third-instar larvae stop feeding in late autumn and overwinter in their third stage in the soil at around 5-10 cm depth. The pupation stage starts in April and is short, less than 1 month, larvae pupating in small earthen cells after a few weeks of feeding in early spring. This lifecycle highlights seasonal activity, including the timing of emergence, adult stages and larval stages. It reveals when and for how long the pest is active in each of its life stages and provides critical information for pest management strategies (Simões and Martins 1985). The monitoring and surveillance of invasive species, as well as the estimation of the damage and the extent of the impacts of *P.japonica* populations in various regions, are crucial. These data contribute to the attempts to evaluate the environment regarding invasive species management. Research on Japanese beetle population dynamics is necessary for periodic monitoring to gain an understanding of its biology, including behaviour and population changes and for which pest management approaches will be improved through insights into behavioural aspects of its distribution, abundance and life cycle (Régnière et al. 1981, Vittum 1986, Potter and Held 2002).

*Popilliajaponica* is considered one of the most harmful invasive species for agriculture worldwide (Tayeh et al. 2023), being able to feed on more than 414 plants (138 main host plants and 276 secondary host plants) and is one of the most important pest insects in agricultural areas. The economic cost to agricultural areas due to the presence of *P.japonica* can be significant, resulting in significant yield losses (Potter and Held 2002). A study conducted in wine grape vineyards has highlighted that the quality of grapes could be compromised as beetle density and defoliation increased, especially with a significant increase in acidity levels and a negative impact on yield (Ebbenga et al. 2022b). These data highlight the detrimental effects of *P.japonica* infestations on crops and emphasise the need for effective monitoring and control measures.

**Figure 1.**
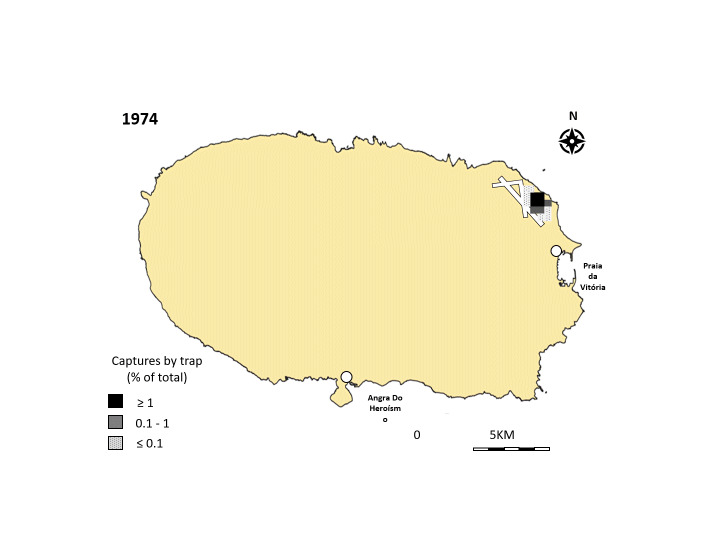
Diffusion of *Popilliajaponica* in Terceira Island (Portugal) from 1974 to 1989. Data are based on the number of adults captured annually by traps. Data and figures are adapted from Martins (1996).

Since 1974, after the beetle arrived on Terceira Island, adult populations have been monitored using traps with a sexual pheromone of *P.japonica* [(Z)-5-(Z-Decenyl) dihydro-2 (3H)-furanone (Tumlinson et al. 1977) and floral lure traps placed across the Island. The traps were set from the middle of June to the middle of September and adults trapped were collected two times per week. These traps allowed us to evaluate the geographical extension and densities of the pest in Terceira. The initial data revealed that the pest was initially established in the airport zone spreading in a circular pattern, with a decreasing population density and a dispersion from the infestation areas towards inland areas. In 1985, the *P.japonica* had spread and infested half of Terceira Island and, in 1989, 16 years after the first record, the pest had occupied all the available space (Fig. 1) (Martins 1996).

Meanwhile, in 1985, a contingency plan was drawn up to establish protective measures to prevent the spread of *P.japonica* to the Madeira Archipelago and the Portugal mainland (Decreto Legislativo Regional № 11/85/A, de 23 de Agosto). Later, the plan was re-defined to comply with European Union legislation, drawing particular attention to categorise this insect as a priority pest (Health (PLH) et al. 2018, Hamilton et al. 2018). Although preventative measures have been applied, the pest has spread to other islands over the years and, currently, eight of the nine islands of the Azores Archipelago are infested. In 1996, the Japanese beetle was detected in Faial; in 2003, São Miguel; in 2006, Pico; in 2007, Flores; in 2011, São Jorge; in 2013, Corvo; and in 2017, Graciosa. Only Santa Maria has not yet recorded the pest's presence (Simões and Martins 1985, Martins et al. 1988, Vieira 2008).

Monitoring of *P.japonica* beetles has been carried out using Ellisco traps baited with synthetic pheromones and floral lures. This monitoring can be used to understand the beetle population's size, distribution and spread, through mass trapping. Long-term monitoring data on the presence of *P.japonica* in agricultural areas is needed to fully understand its impacts and to develop strategies for controlling this pest (Health (PLH) et al. 2018, Hamilton et al. 2018).

Ellisco traps are used for monitoring and limiting the spread of *P.japonica* populations. Together, they include pheromone traps, visual surveys and aerial surveillance. Using these monitoring systems, the researchers can detect and trace the outbreak of *P.japonica* populations to identify high-risk areas in the early stage and take measures for control on time (Piñero and Dudenhoeffer 2018).

The *P.japonica* introduction in Europe has drawn attention to the crop and horticultural industries because of the extensive damage it can cause to many crops and plants (Poggi et al. 2022, Gotta et al. 2023). The authorities and researchers are developing effective pest management techniques to eradicate the Japanese beetle population from continental Europe. The most important method implemented for population control of *P.japonica* is the use of pesticides, but pesticides are harmful to humans and the environment. We require innovative strategies such as integrated pest management, which involves multiple methods for dealing with Japanese beetles like biological control, cultural practices, monitoring and trapping (Straubinger et al. 2023, Borner et al. 2023). The implementation of tough regulations and quarantine measures to stop invasions of *P.japonica* by conducting surveys on beetle numbers and distribution and through targeted control measures that include destroying infected plants and the use of natural predators and insecticides particularly made to control these species. Controlling *P.japonica* spread in Europe is a complex process that requires a multifaceted approach (Poggi et al. 2022). This involves a collaboration of stakeholders, including government agencies, researchers, farmers and the public, which will help raise awareness of the invasive Japanese beetle and promote sustainable agronomic and gardening practices to prevent the spread of this pest. Along with this, the research strives to make biology, behaviour and host plant resistance information available to aid in developing effective management strategies (Borner et al. 2023).

The control and reduction of of *P.japonica* in Europe needs a regulatory framework, surveillance and monitoring, targeted control methods and public participation to manage and reduce the pest densisties on European soil successfully. Efforts to slow the spread of *P.japonica* in Europe have been conducted by adopting integrated pest management strategies, stringent regulations and quarantine measures and joint actions of the various stakeholders (Poggi et al. 2022). Nevertheless, it is worth noting that no single control strategy could eliminate the pest; thus, integrated pest management systems that integrate various control methods are usually advocated (EPPO 2016).

Knowing the Japanese beetle's population densities at an early stage is important because it helps to ensure timely and effective pest control for agricultural and ornamental plants (Ebbenga et al. 2022a). Furthermore, surveillance monitoring systems are used to control and monitor the introduction of this invasive species.

## General description

### Purpose

To make data available on the population dynamics of *Popilliajaponica* Newman, 1838 (Coleoptera, Rutelidae) in the Azores, obtained in a long-term monitoring programme.

### Additional information

The Japanese beetle, known as *Popilliajaponica* Newman, 1838 (Coleoptera, Rutelidae), made its way to the Azores in 1970. In 2019, the existing monitoring programme in the Azores was integrated into a Portuguese National Action Plan for addressing *P.japonica*. This plan was elaborated by the national Directorate-General for Food and Veterinary and is grounded in legal frameworks, including Commission Implementing Regulation (EU) № 2019/2072 of 28 November 2019, Commission Delegated Regulation (EU) № 2019/1702 of 1 August 2019 and national Decree-Law № 67/2020 of 15 September 2020 that aims to ensure adherence to protective measures against plant pests.

The "Contingency Plan for *Popilliajaponica* (Newman, 1838)" aims to establish preventative measures to prevent the introduction and proliferation of this insect on mainland Portugal and the autonomous region of Madeira. The objective is a prompt response that aligns with the existing European Union regulations and national legislation concerning the priority categorisation of Japanese beetles requiring regulation.

The European laws mentioned within the scope of the Contingency Plan comprise Commission Implementing Regulation (EU) № 2019/2072, setting out consistent conditions for protection against plant pests, along with Commission Delegated Regulation (EU) № 2019/1702, which identifies a list containing priority pests, including *Popilliajaponica.* These regulations supplement the Regulation (EU) № 2016/2031 of the European Parliament and of the Council of 26 October 2016, focusing on defensive measures to combat plant pests (DGAV 2021).

## Project description

### Title

Long-term monitoring data on populations of *Popilliajaponica* (Newman 1838) across the Azorean Islands

### Personnel

Project leaders: Mário Brum Teixeira, António Onofre Soares, Lucas Lamelas-Lopez, David H. Lopes, José Adriano Mota, Paulo A. V. Borges and Nelson Simões.

Team members: Lara Aguiar (Corvo), Ângelo Duarte, Luís Rego, Luís Souto, Luis Goulart (Faial), Ivan Castro, Rigoberto M. Gomes (Flores), Jaime Ferreira, Isabel M. Goulart (Graciosa), José Bettencourt Gaspar, Vasco Paulos, Raúl Jorge, André Silveira (Pico), Isabel Mendes (Santa Maria), Gabriel Calado (Flores until 2020 and then Santa Maria), Catarina Cabeceiras, Paulo Silveira, Jessica Machado (São Jorge), José Henrique Silva, Hilário C. Arruda, Fábio M. B. Carvalho, Vítor Vicente, José Adriano Mota (São Miguel), Cristina Moules, Dulce Fernandes and Gonçalo Ribeiro (Terceira).

External Consultants: Secretaria Regional da Agricultura e Alimentação, Veterinária e Alimentação Direção Regional da Agricultura, Direção de Serviços de Agricultura.

Parataxonomists: Paulo A.V. Borges and all the team members.

Darwin Core Dataset Management: Mário Brum Teixeira, Lucas Lamelas-López and Paulo A.V. Borges.

### Study area description

The study comprises all nine islands of the Azores Archipelago, which is located in the Northern Atlantic Ocean (roughly at 38°43'17''N 27°13'14''W) and is formed by nine islands of volcanic origin and several small islets, divided into three main groups: the Western Group (Flores and Corvo), the Central Group (Faial, Pico, São Jorge, Graciosa and Terceira) and the Eastern Group (São Miguel and Santa Maria). The Azores are of volcanic origin and have a mild, oceanic climate with relatively stable temperatures and high humidity throughout the year, occasional rainfall and the possibility of fog and persistent winds, mainly during winter and autumn.

### Design description

The sampling programme included the installation of numbered Ellisco traps baited with two different attractant types (pheromone and floral lure). The traps contained a rubber capsule with synthetic pheromone and a small pad with a floral attractant that lures the adults of *Popilliajaponica*. The synthetic sexual pheromone active ingredient (R, Z)-5-(1-decenyl) dihydro-2 (3H)-furanone mimics the aroma of the female-produced pheromone beetles to attract and catch male adults. The two trap attractants were replaced every six weeks. The traps were available from April to November of 2008 to 2023, matching the emergence and the end of the flight period of *P.japonica* adults. A total of 11,897 unique traps were set along the islands over these 16 years, an average of 744 traps per year in the Azores Archipelago (Table 1). In Corvo, approximately 15 traps were set on average annually, while in Faial, 47 traps per year were set. In Flores, the average was 99 traps per year, while Graciosa saw around 62 traps annually. Pico registered the highest average with 193 traps per year, followed by São Jorge with 108 traps annually. Terceira recorded an average of 88 traps per year, São Miguel had around 98 traps per year and Santa Maria saw an average of 34 traps per year. The specimens of *P.japonica* were collected from traps weekly in the infested areas. The collected individuals were identified and the number was recorded by trap number. Besides monitoring, in the most infested places, the traps were used to measure *P.japonica* control (massive capture). These traps allowed millions of adults to be trapped over the years, reducing the spread of the pest and reducing damage to crops and cultures. Additionally, phytosanitary sheet reports were elaborated and provided to technicians and farmers to inform them about pest identity and its population dynamics and spread.

### Funding

This investigation was supported by the project IPM-Popillia: Integrated Pest Management of the Invasive Japanese Beetle, *Popilliajaponica* (grant no. H2020-EU. 3.2.1.1. / ID: 861852). M.T. and A.P. were hired by the project and J.F. received a research fellowship from the IPM-Popillia project. H.M. works as a researcher in the CBA centre, financed by Pluriannual FCT-IP – Programmatic Component – Ref. UIDP/05292/2020. The student M.C. collaborated with the project under the Erasmus+ Mobility for Traineeships programme, from the University of Girona, Faculty of Sciences, Spain. PAVB work was financed by the projects Portal da Biodiversidade dos Açores (2022–2023) – PO Azores Project – M1.1.A/INFRAEST CIENT/001/2022, Azores DRCT Pluriannual Funding (M1.1.A/FUNC.UI&D/010/2021-2024) and FCT-UIDB/00329/2020-2024 – DOI 10.54499/UIDB/00329/2020 (Thematic Line 1 – integrated ecological assessment of environmental change on biodiversity). The monitoring of this pest was undertaken by the operational services of the Secretaria Regional da Agricultura e Alimentação and is always supported by its own budget.

## Sampling methods

### Study extent

The monitoring programme was conducted on the nine islands of the Azores Archipelago. The traps were placed in different locations, taking into consideration the putative: i) location of the entrance and ii) spread routes of the pest. The putative entrance places include airports and ports. After pest arrival, several traps were placed near urban places or houses in agricultural areas, including crops and orchard types, such as maize, plum, banana, potato, coffee, chestnut, fig, orange, several citrus, apple, strawberry, vine, olive and mainly in pastures.

### Sampling description

Adult prospection: the monitoring of *P.japonica* was done through regular observation in infested areas using Ellisco traps. In each trap, a double attractant composed of a capsule of pheromone (a sexual attractant) and a diffuser of floral attractant was placed and replaced every six weeks. In this dataset, the monthly captured are reported. When the number of insects was very high, its calculation was done indirectly through weighing. The average weight of each adult was often measured, as its value varies with the time of year, the type of vegetables eaten and the location (DRDA 2009).

### Quality control

All collected individuals were identified by expert taxonomists in the laboratory.

### Step description

This dataset presents the monthly records as a rule and, depending on the topography of the site, it was sought that the distance between traps is 50–100 metres. The traps were installed from the end of March to early April so that they were already available at the beginning of May, shortly before the emergence of the first adults. The presence of the traps in the field was maintained on average until the end of November.

## Geographic coverage

### Description

The following delineates the area extent, maximum altitude and isolation distance to the near fragment/island of each island: São Miguel, the Archipelago's most extensive island, has an area of 757 km² and its topography culminates at an altitude of 1,103 m above sea level. Its distance to the nearest island, Santa Maria is 97.53 km. In comparison, the Island of Santa Maria has a total land area of 97 km², with its highest geographical point reaching 857 m. Terceira Island, with a covering area of 402 km², exhibits an elevation of 1,023 m and its nearest island (São Jorge) is at 71.67 km of distance. Graciosa, which is relatively smaller in size, covers an area of 61 km^2^, with its highest point reaching 402 m above sea level. It is located 45 km away from the nearest island, São Jorge. São Jorge spans 246 km^2^ and reaches a maximum altitude of 1,053 m. Its closest neighbour is Pico Island, at 32.42 km.

With a land area of 447 km^2^, Pico Mountain is the highest point in all of Portugal and it rises to 2,351 m above sea level. Faial Island, featuring 173 km² of area and its highest point of 1,043 m, is 34.26 km away from Pico Island. Furthermore, Flores Island represents an area of 143 km^2^, the highest point of the island is 914 m above the ocean. The nearest island is Corvo, which is 30 km away. Corvo is the smallest of the nine islands and has an area of 17 km², while its greatest altitude is 718 m above sea level. These geospatial metrics not only define the intrinsic attributes of the Azorean Archipelago, but also act as sources of data for ecological, climatological and geological studies.

### Coordinates

36.77409 and 39.96028 Latitude; −31.39892 and −24.85107 Longitude.

## Taxonomic coverage

### Description

*Popilliajaponica* Newman, 1838

### Taxa included

**Table:** 

Rank	Scientific Name	Common Name
species	Popilliajaponica	Japanese Beetle
order	Coleoptera	Beetle

## Temporal coverage

**Data range:** 2008-4-01 – 2023-11-30.

### Notes

Sixteen years of coverage with yearly 5-month intervals and a study interval from April to November 2008 – April to November 2023.

## Usage licence

### Usage licence

Other

### IP rights notes

Creative Commons Attribution License (CC BY 4.0)

## Data resources

### Data package title

Monitoring populations of *Popilliajaponica* Newman, 1838 over 16 years in the Azorean Islands

### Resource link

https://doi.org/10.15468/gk6p48

### Alternative identifiers

https://www.gbif.org/dataset/d946f5cd-70a9-4a0d-84a1-15bab6b2f552

### Number of data sets

1

### Data set 1.

#### Data set name

Monitoring populations of *Popilliajaponica* Newman, 1838 over 16 years in the Azorean Islands

#### Data format

Darwin Core Archive

#### Character set

UTF-8

#### Download URL

http://ipt.gbif.pt/ipt/archive.do?r=popillia_azores&v=1.6

#### Data format version

version 1.6

#### Description

The dataset is available on the Global Biodiversity Information Facility platform, GBIF. The following data table includes records of adults of *P.japonica* on all Azorean islands when the species occurs. The dataset submitted to GBIF is structured as a sample occurrence dataset. The data in this sampling resource have been published as a Darwin Core Archive (DwCA), which is a standardised format for sharing biodiversity data as a set of one or more data tables. The core data file contains 95,176 records (occurrence ID) of *Popilliajaponica*. This IPT (Integrated Publishing Toolkit) archives the data and, thus, serves as the data repository. The data and resource metadata are available for download from Teixeira et al. (2024).

**Data set 1.** 

Column label	Column description
ID	An identifier for the set of data. May be a global unique identifier or an identifier specific to a collection or institution.
type	Type of the record, as defined by the Public Core standard.
licence	Reference to the licence under which the record is published.
institutionID	The identity of the institution publishing the data.
institutionCode	The code of the institution publishing the data.
datasetName	Name of the dataset.
basisOfRecord	The nature of the data record.
occurrenceID	Identifier of the record, coded as a global unique identifier.
organismQuantity	A number or enumeration value for the quantity of organisms.
organismQuantityType	The type of quantification system used for the quantity of organisms.
lifeStage	The life stage of the organisms captured.
establishmentMeans	The process of establishment of the species in the location, using a controlled vocabulary: 'native', 'introduced', 'endemic', 'indeterminate'.
occurrenceStatus	A statement about the presence or absence of a dwc: Taxon at a dwc terms:Location.
occurrenceRemarks	The reference number of the Ellisco trap that was given by the Regional Azorean services.
eventID	An identifier for the set of information associated with a dwc:Event (something that occurs at a place and time). May be a global unique identifier or an identifier specific to the dataset.
eventDate	Date or date range the record was collected.
year	Year of the event.
month	Month of the event.
samplingProtocol	The sampling protocol used to capture the species.
sampleSizeValue	The number of trap units used per eventID.
sampleSizeUnit	The sample size unit refers to an Ellisco trap.
samplingEffort	The number of effort in traps used in the 30 days of capturing, one trap per day was during 30 days.
eventRemarks	Traps ID number and its island and location identifier "municipality".
islandGroup	Name of archipelago region “oriental”, “central” or “ocidental”.
island	Name of the island.
country	Country of the sampling site.
countryCode	ISO code of the country of the sampling site.
municipality	The full, unabbreviated name of the next smaller administrative region than country (city, municipality etc.) in which the dwc terms:Location occurs.
locality	The specific description of the place; less specific geographic information can be provided in other geographic terms (dwc:higherGeography, dwc:continent, dwc:country, dwc:stateProvince, dwc:county, dwc:municipality, dwc:waterBody, dwc:island, dwc:islandGroup).
minimumElevationInMetres	The upper limit of the range of elevation (altitude, usually above sea level), of the Ellisco traps in metres.
maximumElevationInMetres	The upper limit of the range of elevation (altitude, usually above sea level), of the Ellisco traps in metres.
decimalLatitude	The geographic latitude (in decimal degrees, using the spatial reference system given in geodeticDatum) of the geographic centre of a Location.
decimalLongitude	The geographic longitude (in decimal degrees, using the spatial reference system given in geodeticDatum) of the geographic centre of a Location.
geodeticDatum	The ellipsoid, geodetic datum or spatial reference system (SRS), upon which the geographic coordinates given in decimalLatitude and decimalLongitude are based.
coordinateUncertaintyInMetres	Uncertainty of the coordinates of the centre of the sampling plot in metres.
coordinatePrecision	A list (concatenated and separated) of maps, gazetteers or other resources used to georeference the Location, described specifically enough to allow anyone in the future to use the same resources.
georeferenceProtocol	A description or reference to the methods used to determine the spatial footprint, coordinates and uncertainties.
dateIdentified	The date on which the subject was determined as representing the Taxon.
identificationRemarks	Reference number "A00234 " corresponds to Popillia Japonica in the Azorean Biodiversity Portal.
scientificName	Complete scientific name including author and year.
kingdom	Kingdom name.
phylum	Phylum name.
class	Class name.
order	Order name.
family	Family name.
genus	Genus name.
specificEpithet	Specific epithet.
taxonRank	Lowest taxonomic rank of the record.
scientificNameAuthorship	Name of the author of the lowest taxon rank included in the record.

## Additional information

Over these 16 years, the Azorean Regional Agriculture Services have installed Ellisco traps, for a total of 11897 unique traps or an average of 744 traps per year. We collected and revised the adult capture data to make it available to the scientific community. With these data, we prepared and published a dataset in GBIF (Teixeira et al. 2024), with 95,176 occurrences. With the current data, the occurrence of *P.japonica* in the Azores is confirmed on eight islands, being absent only on the Island of Santa Maria. The most recent updated list of the Azorean arthropods (Borges et al. 2022) does not list the species for Corvo, Graciosa and São Jorge. For this reason, our current contribution includes those islands of the Archipelago where the species is distributed.

**Annual captures of** ***P.japonica*** **adults**

**Figure 2.**
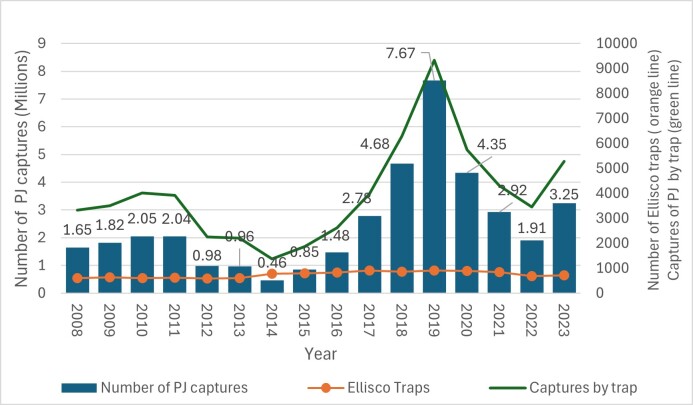
Total number of captures of *P.japonica* (Pj) by year, total number of Ellisco traps installed and number of captures of *P.japonica* by trap in the Azores Archipelago, from 2008 to 2023.

The data in Fig. 2 present the total number of adults of *P.japonica* trapped from 2008 to 2023 revealing a variable annual fluctuation in the number of adults, which could be an indication of a variable activity of the pest. The highest capture rate occurred in 2019, with approximately 7.67 million individuals. In contrast, the lowest capture rate was recorded in 2014, with approximately 0.46 million beetles. Notable peaks also occurred in 2017 and 2018, reaching approximately 2.78 million and 4.68 million individuals, respectively. These changes may be attributed to a combination of factors, including effective pest control measures, environmental factors such as temperature and humidity and natural variations in beetle life cycles, although, in the Azores, the *P.japonica* densities variations and correlation with these factors remains unknown.

**Trap number and** ***P.japonica*** **densities**

The number of Ellisco traps installed on all islands during the same period varied from a minimum of 594 in 2012 to a maximum of 912 in 2019, with an average of 743 ± 124 traps per year (Fig. 2). An overall increase in the number of traps installed over the past 16 years suggests an effort to intensify the monitoring programme and the control efforts against the Japanese beetle infestation.

Both the total number of *P.japonica* captured and the sampling effort (number of Ellisco traps installed) show a gradual increase over the 16-year period. From 2012 to 2015, the number of adults captured declined, despite a significant increase in the number of traps installed (more than 207 traps were installed from 2012 to 2015), suggesting a population outbreak that has not been fully explained (Fig. 2). In 2016, another significant outbreak in captures occurred, but in line with the high number of traps installed, suggesting a possible correlation between these two factors. From 2017 to 2020, the data reveal that, despite the number of traps installed remained mostly the same (average variation of 19 traps per year), the number of captures recorded was about 4.89 million (from 2017 to 2019), followed by a reduction of 3.32 million between 2019 and 2020. This result suggests again that the variation in capture rates was not only correlated with the number of traps.

Overall, while an increase in traps sometimes correlates with higher captures (2016–2017, 2018–2019 and 2022–-2023), the relationship is not straightforward in other years (2012–2015 and 2017–2018), that is, the number of captures does not increase proportionally with the number of traps, indicating that other factors such as pest control management, environmental conditions, trap placement efficiency, trap effectiveness, beetle behaviour changes or densities are involved (Fig. 2).

**Total captures of** ***P.japonica*** **by island**

The number of adults captured per square kilometres varied between the islands regarding their total area and the number of *P.japonica* adults captured during these 16 years of monitoring. The highest average number of adults per square kilometre was registered in the islands of São Jorge, Corvo and Pico with an average of 39828, 35219 and 33433 captures per km^2^, respectively. The Islands of Faial, Flores and Terceira presented 28702, 24308 and 10141 captures of *P.japonica* per km^2^, respectively. The lowest captures were registered in the Islands of São Miguel with 2723 captures per km^2^, in Graciosa with 145 captures per km^2^ and, in Santa Maria, no capture was registered until 2023.

**Figure 3.**
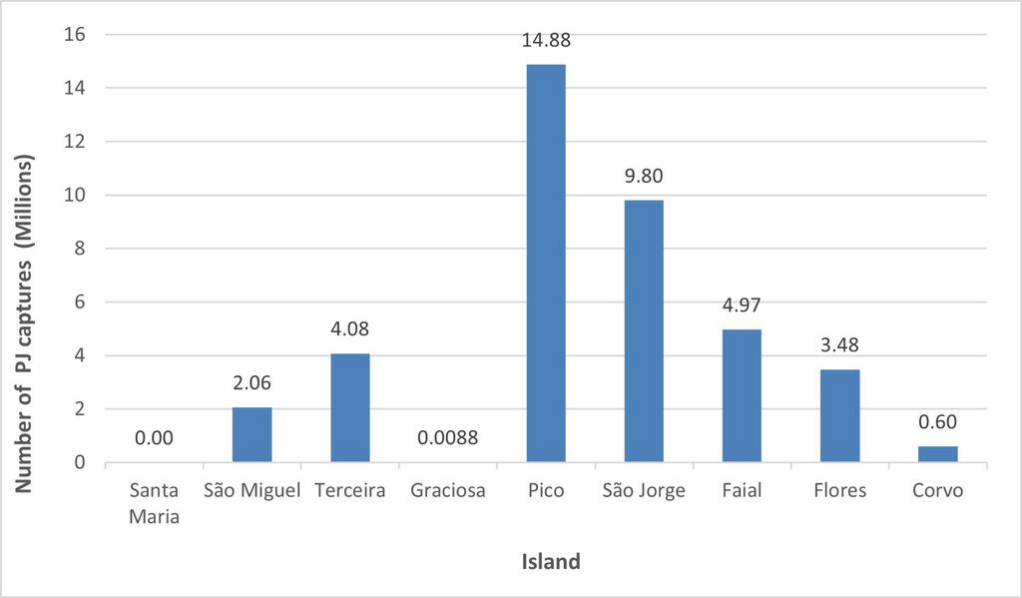
Total number of captures of *P.japonica* (Pj) by islands, from 2008 to 2023.

Fig. 3 presents the total number of *P.japonica* captured from 2008 to 2023 on different islands in the Azores (Fig. 3). The results reveal variations amongst the islands. The highest number of *P.japonica* captured occurred in São Jorge and Pico Islands with approximately 9,797,635 and 14,877,890 individuals, with an average capture per trap of 5696 and 4810 individuals, respectively (Teixeira et al. 2024).

This finding suggests that Pico and São Jorge Islands provide the most suitable habitats for *P.japonica*, potentially due to factors such as climate, vegetation or landscape. Faial and Terceira Islands followed closely, with 4,965,435 and 4,076,831 captures, respectively and an average of 6,621 and 2,904 individuals per trap. These numbers indicate that *P.japonica* also thrives in these locations, although to a lesser extent than on the Pico and São Jorge Islands.

In the remaining islands, including Corvo, São Miguel and Flores, the number of captures varies between 598,719 in Corvo, 2,061,093 in São Miguel and 3,476,059 in Flores, with average captures per trap of 2,444, 1,321 and 2,185 individuals, respectively. In contrast, Graciosa Island presented the lowest number of captures, with 8,853 individuals recorded, with an average capture per trap of nine individuals. These variations suggest that the suitability of these islands for *P.japonica* varies, with some providing more favourable conditions than others.

Notably, Santa Maria Island had no captures recorded during the study period. The absence of *P.japonica* may be due to the island's unique environmental characteristics, like a warmer and drier climate, which contributes to greater aridity on the land and dry vegetation or the presence of competing species that outcompete *P.japonica* for resources (Borges 1999, Azevedo 2001).

**The monthly** ***P.japonica*** **captures**

**Figure 4.**
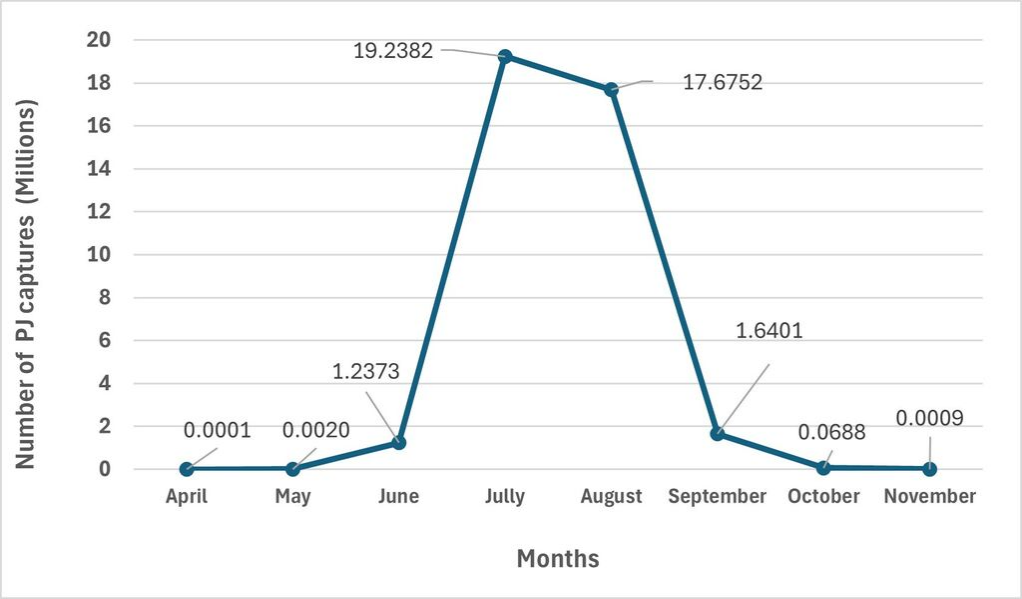
Total number of *P.japonica* (Pj) captured per month, from 2008 to 2023.

During this 16-year study, the results reveal that adult *P.japonica* is most abundant and active during the summer months of July and August, with the highest captures occurring during these periods (Fig. 4).

The number of individuals collected throughout the year strongly varies and reflects the dynamics of its life cycle. In July, approximately 19,238,190 individuals were captured, while in August, the number was around 17,675,170 individuals. This aligns with the typical life cycle of the species, where adults emerge from the soil in late spring to early summer and remain active for several weeks (Simões and Martins 1985). Adults of *P.japonica* lay eggs in the soil during July and August, based on their flight activity during these months. The eggs hatch into larvae (grubs) and feed on plant roots until late autumn or early winter. The lower capture rates in September through November suggest that adult activity declines after the peak occurs in the summer. During September, the captures were around 1,000,000 individuals, while in October and November, the numbers dropped to 68,831 and 860, respectively.

*P.japonica* larvae remain dormant in the soil throughout the winter months. The very low capture rates in April and May indicate that adult emergence from the soil likely occurs in late spring or early summer. In April, only 111 individuals were captured and in May, the number increased slightly to 2,006. The life cycle of *P.japonica* is influenced by environmental factors such as temperature, rain and soil moisture. The mild and humid climate of the Azores may allow for a more prolonged adult activity period from May to September when compared to other regions with more extreme seasonal variations (Simões and Martins 1985, Martins 1996, Azevedo 2001).

### Future perspectives

The study aimed to make data available on the population dynamics of *P.japonica* on the Azores, obtained through a long-term monitoring programme. The information on the yearly and monthly captures of *P.japonica* over 16 years on different islands is presented in this study. The data reveal significant variations in the population density of *P.japonica* amongst the islands and throughout the year, providing valuable insights into the distribution, abundance and seasonal dynamics of this species in the Azores. This 16-year monitoring and eradication programme conducted in the Azores Archipelago also provides valuable insight into the distribution, abundance and seasonal dynamics of *P.japonica*, which can be used to develop targeted and efficient management strategies. The maintenance of this long-term monitoring programme is crucial for effective biocontrol and providing information for political decisions on pest control.

## Figures and Tables

**Table 1.** **Table 1.** List of Ellisco traps set by year in Azores and correspondent total captures of *P.japonica* (PJ).

**Year**	**Ellisco traps**	**PJ captures**
**2008**	605	1649077
**2009**	635	1820366
**2010**	601	2053433
**2011**	618	2042223
**2012**	594	982665
**2013**	603	964447
**2014**	785	461680
**2015**	801	848593
**2016**	828	1477943
**2017**	908	2784879
**2018**	862	4676118
**2019**	912	7673312
**2020**	897	4347036
**2021**	848	2924221
**2022**	688	1906140
**2023**	712	3250382
**Total**	11897	39862515
